# Dynamically Quantifying Vocal Fold Thickness: Effects of Medialization Implant Location on Glottal Shape and Phonation

**DOI:** 10.3390/bioengineering12060667

**Published:** 2025-06-18

**Authors:** Charles Farbos de Luzan, Jacob Michaud-Dorko, Rebecca J. Howell, Ephraim Gutmark, Liran Oren

**Affiliations:** 1 Department of Otolaryngology-Head and Neck Surgery, College of Medicine, University of Cincinnati, Cincinnati, OH 45267, USAorenl@ucmail.uc.edu (L.O.); 2 Department of Biomedical Engineering, College of Medicine, University of Cincinnati, Cincinnati, OH 45267, USA; michaujj@mail.uc.edu; 3 Department of Aerospace Engineering, University of Cincinnati, Cincinnati, OH 45267, USA

**Keywords:** stereo-DIC, vocal folds dynamics, recurrent laryngeal nerve paralysis, clinical application

## Abstract

Unilateral vocal fold paralysis (UVFP) can lead to significant dysphonia. Medialization thyroplasty type 1 (TT1) is a common surgical intervention aiming at improving vocal quality by optimally positioning the paralyzed fold to generate the necessary vibrations for phonation. Implants are generally placed through the thyroid cartilage in a sedated patient and positioned either underneath the level of the vocal folds (infraglottal medialization or IM) or at the level of the vocal folds (glottal medialization or GM). Using high-speed three-dimensional digital image correlation (3D-DIC) in an ex vivo canine hemilarynx model, this study explores the impact of implant location, specifically IM versus GM on the pre-phonatory and dynamic vertical thickness, glottal divergence, flow rate (Q), and cepstral peak prominence (CPP) under varying adduction and subglottal pressure conditions. IM consistently increased glottal divergence and dynamic vertical thickness, particularly in under-adducted states (AL1), despite producing lower static thickness than GM. CPP remained unaffected by the implant condition, but Q decreased significantly with IM under AL1, indicating enhanced glottal resistance and closure. These findings suggest that IM may offer superior functional outcomes by restoring divergent glottal shaping and improving vibratory efficiency. This study also introduces a validated method for dynamically quantifying vocal fold thickness and emphasizes the importance of implant depth in medialization thyroplasty strategies.

## 1. Introduction

### 1.1. Unilateral Vocal Fold Paralysis (UVFP)

Unilateral vocal fold paralysis (UVFP) occurs when recurrent laryngeal nerve damage impairs vocal fold function, affecting speech and swallowing. The severity depends on the fold’s position (lateral or paramedian). Since phonation requires proper glottal closure, medializing the paralyzed fold is necessary to restore voice function. Thyroplasty type 1 (TT1) is a commonly used surgical intervention for UVFP. This procedure involves placing a silastic block through a window in the thyroid cartilage to push the immobile fold toward the midline, enabling the vibrations necessary for phonation. Although TT1 often improves vocal quality, it does not fully restore normal voice function, and long-term outcomes can be variable. Anderson et al. [1] reported that 33% of patients require secondary procedures post thyroplasty, highlighting its limitations in achieving durable results. These findings underscore that UVFP management is not a one-dimensional (1D) problem where simply closing the glottal gap suffices. Instead, restoring vocal function requires addressing the complex, 3D vibratory patterns of the glottis. Multiple studies have demonstrated that the vibratory modes and shapes of the vocal folds are highly 3D, as revealed through FSI modeling and experiments [2,3]. This emphasizes the need for a deeper understanding of the biomechanical mechanisms underlying TT1 outcomes to optimize surgical techniques and improve patient treatment.

Type I thyroplasty aims to improve glottal closure by medializing the vocal fold, which can be adjusted intraoperatively to optimize phonation [4]. However, surgical outcomes are also influenced by changes in mucosal wave behavior and vibratory patterns, which depend on multiple biomechanical factors, such as anatomical structure and tissue properties.

### 1.2. Importance of Glottic Shape

The dynamic behavior of vocal folds during phonation is fundamental to voice production and vocal health. Glottal fold vibration arises from the interaction between the intrinsic fold characteristics and the aerodynamic forces in the respiratory tract. Hirano et al. [5] described it as a mucosal wave that travels along the vocal fold, causing an alternation between convergence and divergence in the glottis shape. Understanding the mechanisms underlying the convergent–divergent glottal shapes is crucial. Traditional studies, such as the overview of mucosal wave mechanics by Berke et al. [6] and the analysis of medial surface dynamics in canine vocal folds by Dollinger et al. [7], have provided foundational insights into the biomechanics of voice. Notably, the divergent shape correlates with increased acoustic intensity and vocal efficiency [8,9]. Depending on the divergence angle, intraglottal airflow may separate from the glottal walls, which generates turbulence negative gauge pressure that promotes glottal closure [10,11]. Previous research showed minimal divergence at a low subglottal pressure (P_sub_), which can be assimilated to the lung pressure, and increasing divergence at a higher P_sub_. Indentation techniques in ex vivo canine larynges revealed a vertical stiffness gradient (VSG) at a high P_sub_ [12]. A stiffer infraglottal aspect leads to less lateral travel than the glottal aspect, causing a divergent contour [13]. In the context of TT1 specifically, we previously quantified VSG in a canine model and linked it to the vertical location of the implant [14]. This work demonstrated that infraglottal medialization (IM), which involves implant placement along the inferior aspect of the vocal fold, promotes a greater VSG compared to glottal medialization (GM), where the implant is positioned at the mid-membranous level. These findings suggest that IM may enhance vocal fold dynamics during phonation more effectively than GM.

Early work by Van den Berg [15] mentioned vertical thickness as a factor influencing register shifts, attributing these shifts to changes in vertical thickness—among other controls—under the activation of the laryngeal muscles. The study also speculated that inherent differences in vertical thickness, such as those observed between males and females, could influence the pitch range achievable by an individual when all controllable parameters are exhausted. However, no method for quantifying vertical thickness was proposed at that time. More recently, a review on the topic by Zhang [16] emphasized the critical role of vocal fold vertical thickness in regulating glottal closure patterns and the spectral characteristics of the voice. Zhang’s conclusions were based on numerical simulations using a parametric model of the vocal fold, where vertical thickness was defined as the vertical length of the medial surface, a parameter that could be controlled. These simulations demonstrated that vertical thickness significantly affects the phonation threshold pressure, closed quotient, and spectral measures such as the slope between the first two harmonics (H1–H2) and the cepstral peak prominence (CPP). However, the experimental studies cited in the review did not provide a method for quantifying vertical thickness during phonation in a tissue model. The findings underscored the need for future research to focus on the 3D medial surface shape due to its dominant effect on voice quality and its clinical implications for interventions targeting vocal fold thickness. Initial attempts to measure the thickness measured the vertical extent of the surface within a chosen threshold distance [17]. Later, Lehoux et al. [18] introduced a different parametric integral method for quantifying vocal fold thickness in a static larynx, using a weight function along the inferior–superior axis to account for the vertical distribution of thickness. Farbos de Luzan et al. [19] proposed a methodology for dynamically quantifying vocal fold thickness during phonation in an excised larynx model by integrating the medial–lateral displacement of the medial wall.

### 1.3. Vocal Fold Shape Measurement

From a clinical perspective, the ability to measure the three-dimensional (3D) geometry of a pathological glottis during phonation at high temporal and spatial resolutions would significantly enhance diagnostic and treatment strategies. Glottal vibratory patterns, coupled with acoustic feedback, play a key role in diagnosing voice-related pathologies and tailoring therapeutic interventions. While videostrobolaryngoscopy remains the most widely used clinical tool for assessing glottal dynamics, its reliance on periodic acoustic signals limits its applicability to pathological phonation [20,21]. In healthy speech, the fundamental frequency of the larynx typically ranges from 100 to 200 Hz in adults and up to 300 Hz in children. However, irregular vibrations in pathological voices make phase-locking with a periodic signal unreliable, reducing the utility of this technique.

In research settings, high-speed laryngoscopy [22] and videokymography [23] offer an improved visualization of the pathological vocal fold vibrations. These methods do not rely on periodic signals, enabling the capture of detailed cyclic glottal behavior in irregular phonation. High-speed laryngoscopy typically employs acquisition rates between 2 kHz and 8 kHz to accurately evaluate vocal fold vibration. Interestingly, Birk et al. [24] explored varying frame rates to assess their effect on the subjective evaluations of vocal quality metrics, highlighting the importance of resolution in capturing dynamic behavior.

Despite these advancements, both techniques remain limited to two-dimensional (2D) imaging, requiring significant resources for data acquisition, processing, and analysis. This restricts their ability to provide comprehensive insights into the 3D vibratory dynamics essential for understanding complex glottal behavior. Some techniques were developed after these ones and provided a 3D measurement of the superior surface of the vocal fold, such as depth-kymography [25,26] or digital holographic interferometry [27].

Using a stereoscopic prism–single-camera setup, Dollinger and Berry [2] measured the medial surface dynamics in an excised human hemilarynx, which yielded a spatial resolution of 2 mm and a vertical resolution of 1.7 mm. This resolution was defined by a micro-stitches grid pattern that was created on the medial surface of the hemilarynx. Cameron et al. [28] improved the resolution by applying a speckle pattern on the tissue using black makeup, allowing them to measure the changes in the ex vivo medial shape with implants of varying stiffness. Notably, their study assessed the 3D shape in static models. They emphasized that “measurements of vertical thickness based upon the glottal contact and vibratory areas might be more accurate ways of evaluating the role of implants on VF vertical thickness.” In that study, they measured the 3D shape in static models. Three-dimensional digital image correlation (3D-DIC) is a full-field non-contact optical technique to measure contour, deformation, vibration, and strain on almost any material [29]. The introduction of this technique presented an opportunity to capture and analyze vocal fold vibrations with unprecedented temporal and spatial resolution. Originally introduced in the early 1980s, it started to be used in the biomechanical field in the early 1990s [30]. The planar DIC technique involves tracking a speckle pattern applied to a surface to measure deformation over time. By using two high-speed cameras in a stereoscopic configuration and a calibrated target for spatial co-registration, DIC enables the extraction of full 3D surface contours and motion. This approach captures not only in-plane displacements but also out-of-plane (depth) movement, resulting in a time-resolved 3D vector field of the surface dynamics. The present research uses the enhanced capability of 3D-DIC and provides a deeper understanding of vocal fold dynamics in the context of TT1, thereby offering new avenues for diagnosing and treating voice disorders. Through a detailed examination of both the methodology and the resultant data, we aim to demonstrate how 3D-DIC significantly extends the existing knowledge base and provides a new understanding of vocal fold dynamics. This paper builds on the previously cited works by introducing novel 3D-DIC data that reveals intricate details of the vocal fold surface during phonation, which were previously obscured or inaccessible with other methodologies [7,31,32].

While earlier studies have contributed valuable insights into mucosal wave mechanics, there remains a need for the precise quantification of vertical mucosal wave thickness during phonation. Prior research has often relied on indirect or qualitative assessments, lacking the resolution necessary for a detailed understanding of vocal fold dynamics. In this study, we introduce a novel analytical approach to directly quantify the vertical thickness of the mucosal wave in an excised hemilarynx. This technique enables the precise measurement of dynamic changes in fold thickness throughout the phonatory cycle, providing a quantitative metric that bridges the gap between numerical simulations and experimental data. By systematically measuring the vertical thickness, our approach offers a deeper understanding of its role in phonatory outcomes and highlights the clinical potential for optimizing vocal fold medialization procedures.

In this study, we compare two implant placement strategies for medialization thyroplasty, namely GM vs. IM. We hypothesize that IM will more effectively increase vertical thickness in the paralyzed fold, thereby enhancing the vertical mucosal wave and amplifying the convergent–divergent glottal angle—particularly its divergent component during the closing phase of vibration. To evaluate this, we quantify vocal fold thickness using the weighted integration method described by Lehoux et al. [18]. These findings may inform optimal implant placement along the vertical axis and potentially improve surgical outcomes in patients with vocal fold paralysis.

In the context of this study, we hypothesize that IM will more effectively increase the vertical thickness in the paralyzed vocal fold, which will enhance the vertical mucosal wave and the amplitude of the convergent–divergent glottal angle, more importantly, the divergent portion of it during closing. We also propose to quantify the vocal fold thickness using the previously mentioned method. This way, our findings may offer valuable insights into identifying the optimal implant location along the vertical axis of the fold during medialization thyroplasty, potentially improving surgical outcomes for patients with vocal fold paralysis.

## 2. Materials and Methods

### 2.1. Hemilarynx Preparation

Seven ex vivo larynges (L1 to L7) were excised from healthy shared research Mongrel canines (weight range of 20–22 kgs), following the animal use protocol approved by the University of Cincinnati′s institutional animal care and use committee (IACUC protocol #23-03-28-03). The nominal length of the vibrating membrane of the vocal folds ranged from 13 to 14 mm, measured from the vocal process to the anterior commissure, and the vertical height, which was measured on the static vocal fold, ranged from 3 to 4 mm (from the superior to the inferior edge). Part of the trachea was preserved (about 5 tracheal rings down from the cricoid) to be used as an airflow inlet, and the tissues above the vocal folds were removed. The thyroid cartilage was also cut along the axial plane immediately above the glottal aspect of the vocal folds. The glottal mucosal surface of the free margin of the folds was stitched to the resulting edge of the thyroid cartilage to maintain the larynx’s integrity, without adding any unnatural strain to the tissue.

A hemilaryngectomy was performed by removing the right fold, leaving the trachea intact. The hemilarynx was attached to a transparent Plexiglas plate using a suture at the anterior commissure and two on the thyroid cartilage to secure it in place. Sealing was ensured by a bead of silicone sealant, delineating the contact between the thyroid cartilage and the transparent plate.

The model was mounted on an aerodynamic nozzle, which supplied the subglottis with conditioned airflow at a known flow rate and pressure. The experimental setup was adapted from Oren et al. [33] to hold the Plexiglas plate vertically in the mid-sagittal plane, while an opposed mechanical prong was inserted into the remaining arytenoid cartilage to control the adduction level of the hemilarynx using a three-axis micrometric screw system. This approach enabled the measurement of different adduction levels.

**Figure 1:**
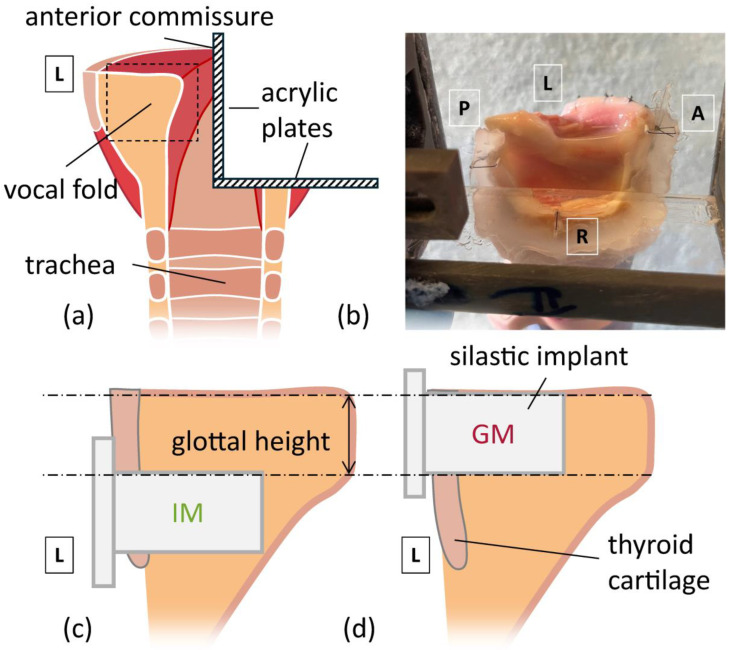
Implant Locations in the hemilarynx. Anatomical directions are labeled (A = anterior, P = posterior, L = left, R = right). (**a**) Cross-section of the mid-coronal plane of larynx post removal of supraglottal tissues, showing internal structure and the wedge formed by acrylic plates. (**b**) picture of the hemilarynx when affixed to the acrylic wedge. (**c**,**d**) are enlarged views from the dashed rectangle in (**a**), depicting (**c**) infraglottal medialization (IM) and (**d**) glottal medialization (GM).

**Figure 2:**
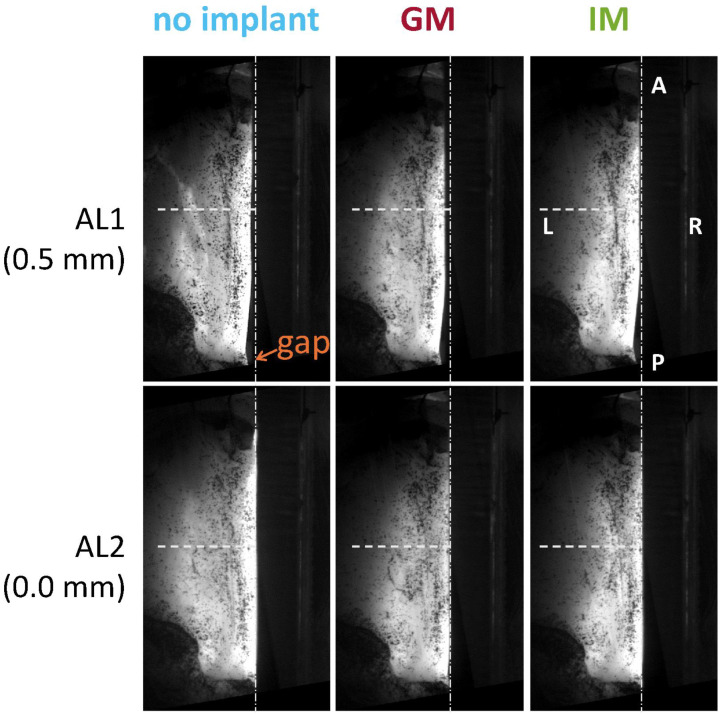
Effect of adduction level on vocal fold positioning; shown in the top view for a representative larynx. Anatomical directions are labeled (A = anterior, P = posterior, L = left, R = right). AL1 represents a less adducted condition than AL2, with a 0.5 mm difference. AL1 produces a visible posterior glottal gap, indicated near the vocal process. The horizontal dashed line marks the mid-membranous plane for spatial reference.

To evaluate the effect of implant location on the medial shape of the glottis, three implant conditions were evaluated: baseline without an implant (no implant), implant at the glottal level (GM), and implant placed in the infraglottic space (IM). Figure 1 shows a schematic of the larynx model setup, with the implant location specified in reference to the glottis height. To evaluate the effect of medialization depth, two levels of adduction were tested. Adduction level 1 (AL1) simulated the case of vocal insufficiency, where the arytenoid of the paralyzed fold was removed 0.5 mm laterally from the mid-sagittal plane using micrometric screws. This induced a posterior glottic gap that is visible in Figure 2. Adduction level 2 (AL2) simulated the medially adducted case, where the vocal process was medialized against the Plexiglas plate.

Phonation dynamics were acquired at low and high P_sub_ conditions, which were independent variables, being 1.40 kPa (SD = 58 Pa) and 1.87 kPa (SD = 57 Pa), respectively. In total, each larynx underwent a series of 12 tests, summarized in Table 1. 

Phonation of the larynges was achieved by supplying air to the aerodynamic nozzle, and the average flow rate (Q) was measured by a Coriolis flowmeter. The time-averaged P_sub_ was measured in the nozzle via a pressure transducer. The pre-phonatory position of the vocal fold was adjusted by adducting it via a three-pronged micrometric screw system. 

Trials were conducted at low and high P_sub_ conditions, which were independent variables, being 1.40 kPa (SD = 58 Pa) and 1.87 kPa (SD = 57 Pa), respectively.

### 2.2. Acoustic Measurements

A 1/4″ multi-field microphone (4961, Brüel & Kjær, Marlborough, MA, USA) was placed 30 cm above the glottis to capture the acoustic pressure during phonation. The data was sampled at a rate of 40 kHz using a PXIe-4492 microphone card mounted in a PXIe-1082 chassis (National Instruments, Austin, TX, USA). The microphone was placed outside the glottal jet path to prevent interference with the hydrodynamic pressures. CPP was derived from the acoustic measurements for comparative analysis, using the methodology described by Hillenbrand et al. [34].

### 2.3. Stereoscopic Digital Image Correlation (DIC)

Two high-speed cameras (Phantom Miro M340) were mounted in an axial plane located at the level of the superior aspect of the vocal fold. They were oriented in a stereoscopic configuration, such that the cameras’ lines of sight (lens axes) were co-planar concurrent with the trachea axis (i.e., the nozzle axis). They were fitted with 2× teleconverters and Zeiss Milvus 2/100M lenses. The angle between the cameras’ axes was 20°. This arrangement allowed both cameras to capture a speckle pattern that was marked onto the surface of the fold through the Plexiglas plate. The speckle pattern was made of graphite powder, which was deposited onto the humidified mucosa via a dry powder spray bottle prior to each experiment. The medial plate was thoroughly cleaned prior to each trial to ensure optimal conditions for optical tracking. In some cases, graphite particles transferred from the tissue surface to the glass during contact. When this interfered with the DIC pattern or reconstruction quality, the corresponding trial was excluded and repeated to avoid artifacts in the analysis. This optical setup yielded a spatial resolution ranging from approximately 77.6 to 89.4 pixels/mm, depending on the experiment.

The larynges’ fundamental frequencies ranged from 78 to 196 Hz, which yielded between 9 and 22 snapshots per glottal cycle. Several phonation cycles were acquired for each phonation test. During the high-speed imaging of the glottic superior surface, the tissues were illuminated by a 527 nm high-speed dual cavity Nd:YLF laser (Amplitude, Bordeaux, France).

### 2.4. Data Processing

For L1 and L2, images were acquired phase-locked to the P_sub_ signal. From L3 to L7, a fixed 2 kHz frame rate was used for improved image quality and system stability. Phase-locking was used initially to enable cycle-averaged analysis but was later discontinued due to the unreliable triggering from the electroglottograph signal in the hemilarynx setup (see Table S1 in Supplemental Materials for the full dataset, including the number of images per cycle produced by the fixed frame rate combined with the variable fundamental frequency of the larynx).

**Figure 3:**
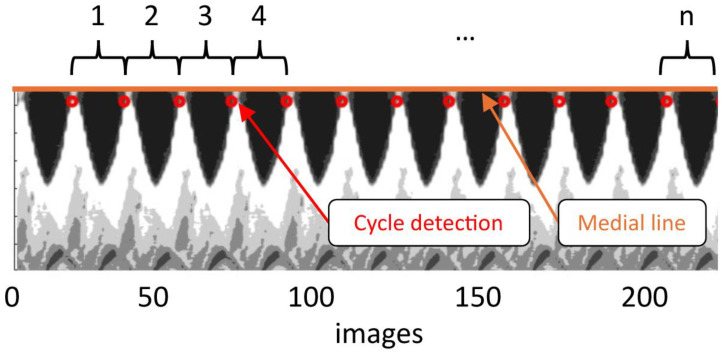
Digital kymogram used for phonation cycle detection in the hemilarynx. Data is extracted from the mid-coronal plane. Each phonatory cycle is identified using an intensity-based threshold (red circles), with θ=0° set at the onset of the closed phase.

The dataset was segmented into subsets of complete cycles, discarding the incomplete cycles at the beginning and the end. Videokymography along the mid-membranous plane was used on the raw images for this purpose [23]. Each cycle was identified by analyzing the contrast of the kymogram and then processed independently (Figure 3). All cycles were averaged to produce a representative mean cycle for each experiment, which together constitute the data that will be discussed in this paper.

**Figure 4:**
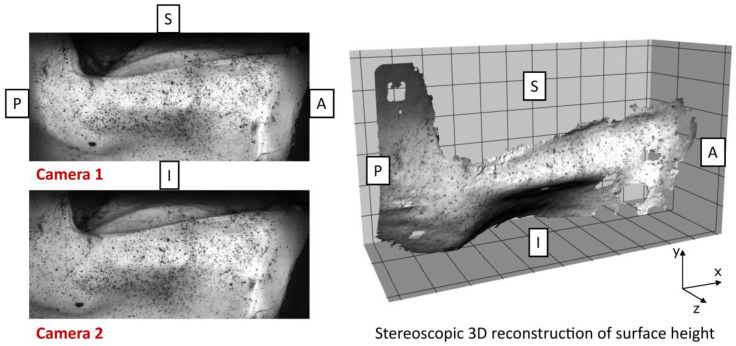
Raw 3D digital image correlation (DIC) reconstruction of the vocal fold medial surface during phonation. Anatomical directions are labeled (A = anterior, P = posterior, I = inferior, S = superior). Left: stereo camera images acquired simultaneously. Right: resulting 3D surface reconstruction. Missing regions indicate areas where speckle pattern quality or tissue deformation exceeded DIC tracking limits. No interpolation was applied to maintain data integrity.

Initial processing of the images was performed with Davis 10.2 StrainMaster software (LaVision GmbH, Göttingen, Germany) with a correlation subset size of 9 pixels and a step size of 3 pixels. The 3D medial shape of the glottis was acquired in its pre-phonatory state and then during phonation (Figure 4).

### 2.5. Statistical Analysis

To assess the effects of implant location, a repeated-measures statistical framework was employed. Each larynx served as its own control, with measurements collected across all combinations of implant condition, AL, and P_sub_. A linear mixed-effects model was used to analyze the outcome variable using pairwise comparisons, with implant location, AL, and P_sub_ specified as fixed effects, and the larynx included as a random intercept to account for repeated measures within subjects [35,36]. *p*-values were adjusted for multiple comparisons using Tukey’s method. The standardized effect size (Cohen’s d) was used for each pairwise contrast to quantify the magnitude and direction of differences between implant conditions. Effect sizes were interpreted as small (|d| ≈ 0.2), medium (|d| ≈ 0.5), or large (|d| ≥ 0.8), providing context for the practical relevance of the observed changes. All analyses were conducted in R software (version 4.4.1), with the significance defined as *p* < 0.05.

## 3. Results

### 3.1. Acoustic and Flow

**Figure 5:**
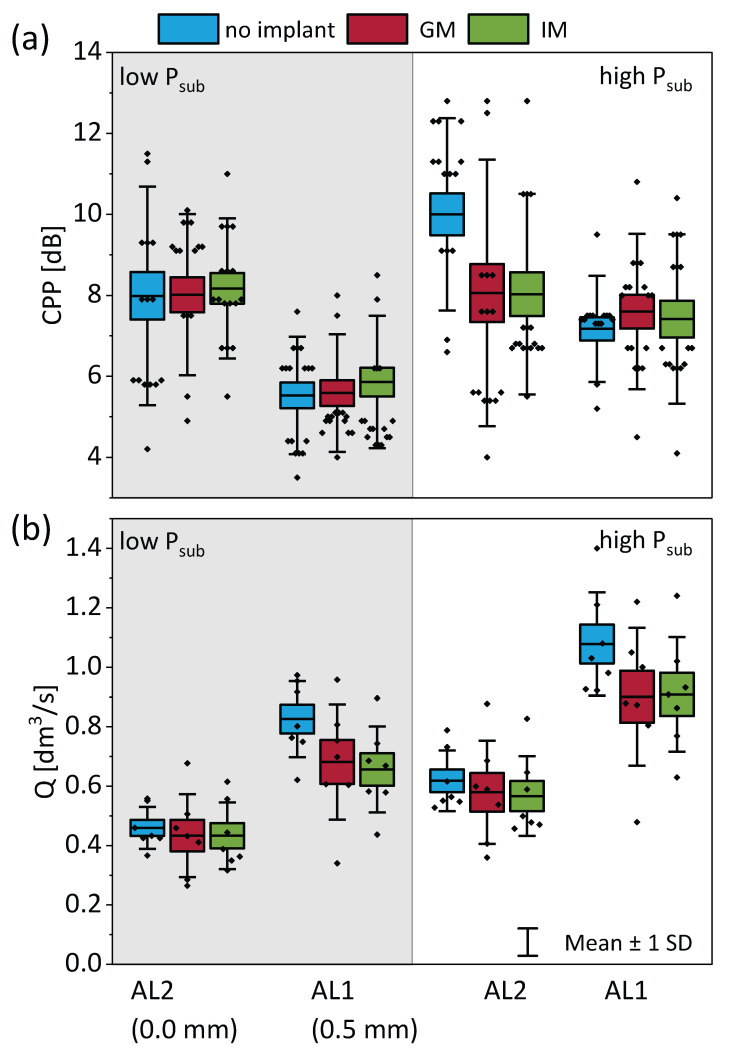
Phonatory outcomes as a function of implant location, adduction level (AL), and subglottal pressure (P_sub_). (**a**) Cepstral peak prominence (CPP) as an indicator of voice quality and breathiness. (**b**) Mean glottal flow rate (Q) measured under each condition.

Medialization had a noticeable effect on phonatory flow characteristics, particularly at lower adduction levels (AL1), as shown in Figure 5a.

The overall effect on Q can be explained using Poiseuille′s law, expressed as

(1) $$Q=\frac{\Delta P}{R}$$

where ∆P is the transglottal pressure (~P_sub_) and R is the glottis resistance. Under constant P_sub_, the implant insertion increased R, which in turn reduced Q. Statistical analysis using a linear mixed-effects model confirmed that both GM and IM implants significantly reduced Q compared to the no-implant condition at AL1 under both low and high P_sub_ (*p* < 0.01), with very large effect sizes (Cohen′s d > 1.7) (Table 2). This reflects the functional improvement in glottal closure and flow resistance when correcting posterior glottal gaps. However, IM did not significantly outperform GM in reducing flow at these conditions. In contrast, at AL2 (higher adduction), neither implant significantly reduced Q compared to the no-implant condition at either subglottal pressure, and effect sizes were small. This suggests that when the vocal folds are already more adducted, additional medialization contributes less to glottal resistance and flow control.

Medialization also affects acoustic characteristics, particularly CPP, which is considered a good indicator of breathiness. Plotting CPP across implant conditions at different adduction levels and subglottal pressures (Figure 5b) revealed higher values with increased adduction (AL2 vs. AL1) and with the presence of an implant compared to no implant. Statistical analysis confirmed this trend only at AL2 under high subglottal pressure, where both the GM and IM implants produced a significant increase in CPP relative to the no-implant condition (*p* < 0.05, Cohen’s d > 1.4), consistent with reduced breathiness. However, no significant difference was observed between GM and IM, suggesting that both implant types improve acoustic outcomes similarly in this context. At other conditions (AL1 and/or low P_sub_), no statistically significant effect of implant location on CPP was found, indicating that implant-mediated improvements in breathiness are more likely when baseline glottal closure is already high, as in AL2.

In summary, medialization, and particularly IM, reliably reduces the mean flow rate by increasing glottal resistance, with the greatest effect seen in under-adducted conditions (AL1).

### 3.2. Dynamic Vocal Fold Measurements

**Figure 6:**
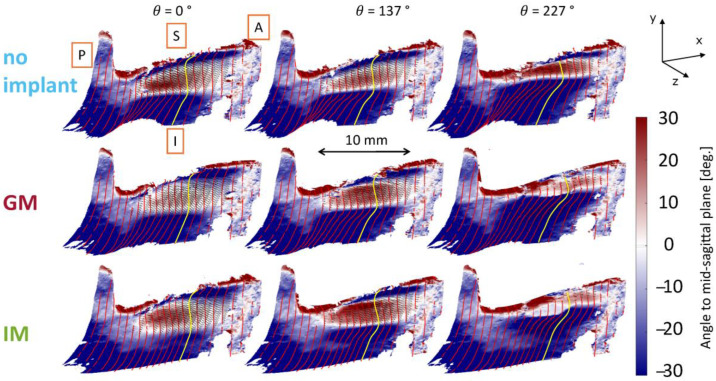
Phase-averaged 3D glottal medial surface shape for larynx L2 during phonation with under-adducted vocal folds (AL1) and three implant conditions: no implant, glottal, and infraglottal. Anatomical directions are labeled (A = anterior, P = posterior, I = inferior, S = superior). Color represents the surface angle relative to the mid-sagittal plane: positive values (red) indicate divergent shapes (opening), and negative values (blue) indicate convergent shapes (closure). The yellow line marks the mid-coronal plane. White regions mark tangency between the vocal fold and the medial plate, where the surface is parallel but not necessarily in full contact or compression.

Data from the DIC analysis provides insights into the difference in the shape of the glottal wall. Figure 6 shows the phase-averaged data of the glottal medial surface for one larynx (L2) at AL1 for the three implant locations.

The 3D medial glottal shape is phase-averaged and plotted over selected phases of the phonation cycle, represented using *θ*. As defined earlier, θ=0° corresponds to the beginning of the closed phase (contact at the inferior aspect of the glottis). The surface is colored by the angle (in degrees) created by the medial surface relative to the mid-sagittal plane (i.e., the transparent plate). A negative angle indicates that the surface faces downward, converging in the airflow direction, while a positive angle represents an upward inclination, diverging in the airflow direction. The mid-coronal plane is highlighted by the yellow line for spatial reference, and the white regions indicate the points of tangency between the medial surface and the medial plate, tracking the inferior margin of the vocal fold. A length scale is included to provide spatial context for the data. These contours qualitatively show that the IM condition exhibits greater divergence in the glottic region, as indicated by the extended red-shaded areas above the inferior glottal margin. This can be seen particularly during the closing phase at *θ* = 137°. GM reduces the glottal gap compared to the no-implant condition, as evidenced by the decreased extent of the red-shaded regions (negative angles indicating divergence) along the medial surface. This effect is most pronounced in the superior region of the vocal fold, where GM results in a steeper medial surface inclination and a reduced opening compared to the no-implant case, particularly during the closing phase.

**Figure 7:**
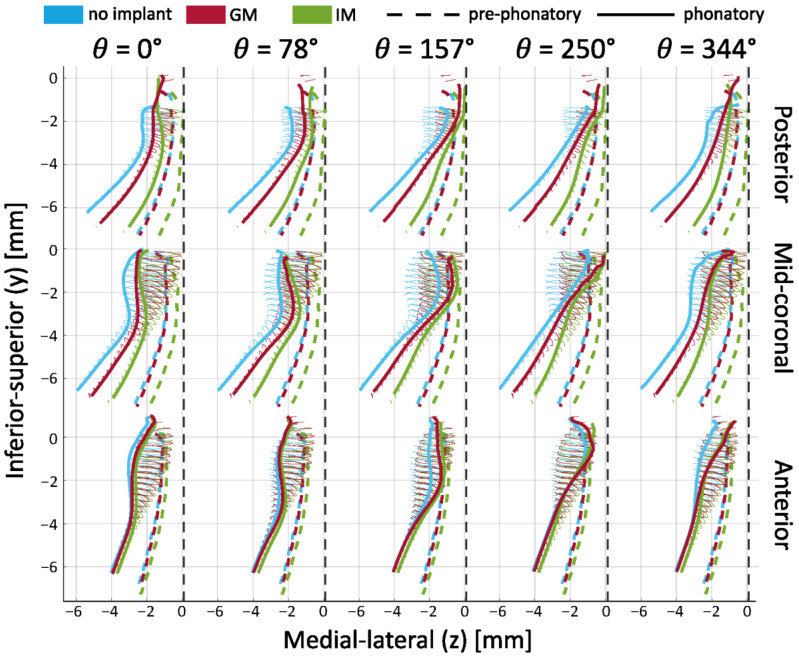
Time-varying medial surface contours during phonation in larynx L2 under low P_sub_ and AL1 (0.5 mm). Solid lines show the medial surface shape at three locations (posterior, mid-coronal, anterior) across different vibratory phases. Dashed lines show the pre-phonatory (static) position. Thin ellipses represent the trajectories of selected surface points during oscillation.

The effect of the implant location is shown quantitatively by plotting the displacement of the points connected by the lines imposed on the contour plots over the (mean) vibratory cycle (Figure 7).

The dashed lines show the pre-phonatory state, further emphasizing the effects of medialization. Specifically, the dashed green line (IM) is closer to the vertical midline, particularly in the infraglottal region. The dashed red line (GM) lies slightly to the right of the blue line (no implant), though this difference appears minor. These pre-phonatory shapes do not necessarily determine the medial surface′s trajectories during vibration (shown with the solid ellipsoids), as the glottis achieves complete closure against the midline in all cases. However, the less medialized condition (blue) consistently remains outside the two implant cases during motion.

The main observations from tracking the medial surface′s trajectories are that the infraglottal shape is narrower and that the divergent angle is larger with IM. The mid-coronal plane demonstrates the highest vibratory amplitude, while the no-implant condition shows limited glottal closure with a persistent posterior gap. Consistent with observations from Figure 6, medialization reduces the glottal gap toward the end of the cycle (e.g., at θ = 157° and θ = 344°). GM and IM display a flat contact area near the midline, absent in the no-implant condition. At θ = 250°, the superior edge separates from the medial plate in the no-implant condition, whereas contact persists in the implant cases. This is indicative of a shorter closed phase in the no-implant case.

IM produced greater glottal divergence and a narrower infraglottal shape during phonation. Both IM and GM improved closure relative to the no-implant case, with IM yielding the most substantial medial surface displacement and contact.

### 3.3. Pre-Phonatory Thickness

The effects of the medialization window location and adduction level on the medial surface shape were assessed using the vocal fold thickness (T), using the methodology introduced by Hampala et al. [17] and refined by Lehoux et al. [18] as

(2) $$T=\int_{L}^{U}w\left(d_{m}\left(y\right)\right)dy$$

where U and L represent the upper (superior) and lower (inferior) bounds of the medial fold surface. The horizontal distance from the most medial point is defined as

(3) $$d_{m}\left(y\right)=z_{m}-z_{c}\left(y\right)$$

The weight function $w\left(d_{m}\right)$ is given by

(4) $$w\left(d_{m}(y)\right)=\left\{\begin{array}{c} 1,\;\mathrm{if}\;d_{m}\left(y\right)\leq \;d_{th}\;\\ e^{-\propto \left(d_{m}\left(y\right)-d_{th}\right)},\;\mathrm{if}\;d_{m}\left(y\right)>d_{th} \end{array}\right.$$

**Figure 8:**
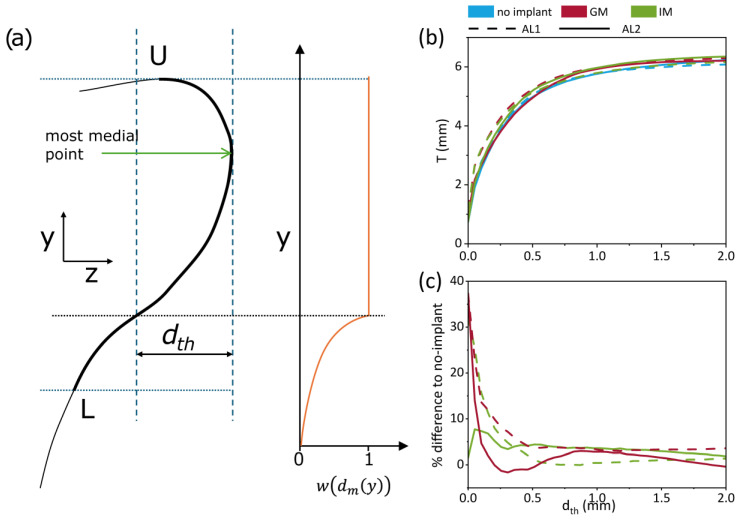
Static vocal fold thickness measured from pre-phonatory geometries. (**a**) Schematic illustrating parameters used in the computation of thickness T adapted from Lehoux et al. [18]. (**b**) Average thickness T across all larynges as a function of threshold distance d_th_. (**c**) Percent difference in thickness between the glottal/infraglottal implant conditions and the no-implant case.

The weight is defined at each vertical location according to the horizontal distance from the most medial point $d_{m}\left(y\right)$ and the threshold distance $d_{th}$, which represents the distance from the most medial point beyond which the weight function starts to decay (Figure 8a). ∝ is a factor determining how fast the weight function decreases. For more details on the methodology, refer to Lehoux et al. [18].

In their study, Lehoux et al. [18] recommended values of ∝=70 and $d_{th}=0.05$ to assess the impact of the parameters on the vertical thickness. This methodology was applied exclusively to the pre-phonatory, static geometries in our dataset, as it does not account for vibratory changes during phonation. In the current study, rather than adopting fixed parameters, we systematically varied $d_{th}$ to evaluate how sensitive the resulting thickness T is to the threshold definition. This approach serves two purposes: it enables comparison across implant and adduction conditions in a consistent way, and it helps identify a practical range of $d_{th}$ values beyond which the measured thickness stabilizes. Figure 8b,c illustrates this parameter sweep, showing that beyond $d_{th}$ ≈ 0.5 mm, the differences between conditions become negligible. Figure 8b shows the impact of $d_{th}$ on the average vocal fold thickness of the 7 larynges, with each line representing a different implant condition. Dashed lines correspond to AL1, while solid lines represent AL2. Generally, increasing $d_{th}$ increases T, regardless of the implant condition, the differences seem minimal and the curves plateau around 6mm. Figure 8c quantifies the subtle changes of IM and GM to the no-implant condition. Differences seem more drastic for small $d_{th}$ and converge between 0 and 10% past $d_{th}=0.5$. The static measurements of vocal fold thickness show that medialization increases thickness, particularly in under-adducted conditions (AL1). GM demonstrates a greater impact on thickness than IM under these conditions (2 to 4% difference). This suggests that implant location has a nuanced effect, potentially driven by how each placement interacts with the fold’s natural anatomy.

These results show that medialization increases vocal fold thickness, especially under low-adduction conditions. In that case, GM produces a slightly greater thickening effect than IM, highlighting the influence of the implant location on pre-phonatory vocal fold shape.

### 3.4. Dynamic Thickness During Phonation

Pre-phonatory vocal fold thickness represents the static medial surface shape before phonation begins. This measure provides insights into how medialization affects the structural configuration of the vocal fold, particularly in terms of medial bulging and the contact area. It is calculated based on the static geometry of the medial surface without considering dynamic deformations. During vibration, the effective thickness of the vocal folds changes due to tissue displacement. Rather than a fixed structural measure, the vertical thickness describes how the medial–lateral motion is distributed across the inferior–superior height of the fold. This requires a different equation because it accounts for dynamic tissue motion, which varies depending on glottal closure, P_sub_, and the vibratory phase.

To assess the vertical thickness (${th}_{V})$ over the vibration cycle, we integrated the medial–lateral displacement of the fold over its height and normalized it by the fold height:

(5) $${th}_{V}=\frac{1}{{\Delta z}_{max}}\int_{0}^{H}\Delta z\left(y\right).dy$$

where $\Delta z\left(y\right)$ is the medial–lateral displacement as a function of the inferior–superior position y, H is the total height of integration, and ${\Delta z}_{max}$ the maximum medial–lateral displacement.

**Figure 9:**
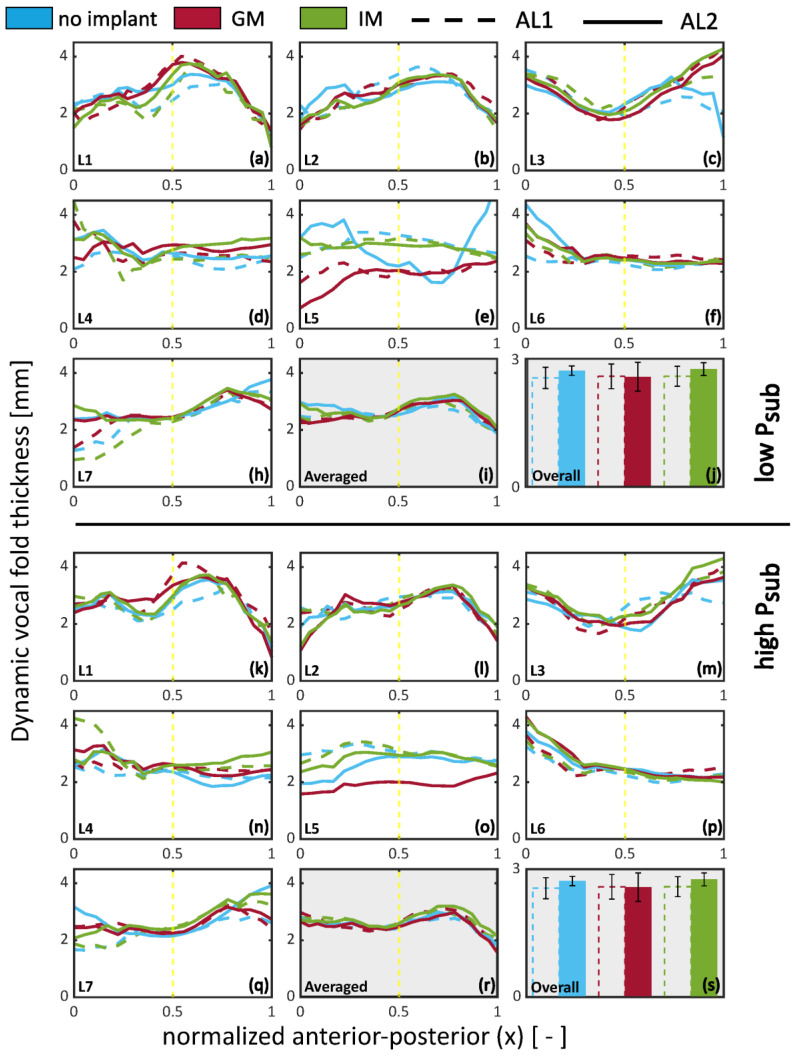
Dynamic vertical thickness (th_V_) of the medial surface during phonation, plotted as a function of anterior–posterior location, implant condition, and adduction level (AL). **Top half**: low subglottal pressure (P_sub_); **bottom half**: high P_sub_. Subfigures (**a**–**h**,**k**–**r**) show individual larynx measurements of vertical thickness across the anterior–posterior axis. Subfigures (**i**,**s**) show the mean vertical thickness averaged across all larynges. Subfigures (**j**,**t**) display the mean thickness averaged along the anterior–posterior axis. The yellow vertical line denotes the mid-coronal reference location. The vertical thickness represents the time-averaged medial–lateral displacement integrated over the phonatory cycle, as defined in Equation (5).

Figure 9 illustrates the dynamic vertical thickness calculated using Equation (5). These values do not correspond to a specific phase angle but instead reflect a time-integrated measure of dynamic thickness, capturing the range of medial–lateral displacement amplitude across the full glottal cycle. Results highlight that the adduction level (AL1 vs. AL2) and implant location impact the vertical thickness. The figure is divided into two halves: the top half gathers low P_sub_ cases, and the lower half gathers the high P_sub_ cases. For each condition, subfigures (a)–(h) and (k)–(q) show the vertical thickness as a function of the normalized anterior–posterior location. Subfigures (i) and (r) show the mean thickness averaged across all larynges, while subfigures (j) and (s) show the mean thickness averaged along the anterior–posterior axis. The range for the mean vertical thickness (2 to 2.7 mm) aligns with the range for the mean values calculated for the pre-phonatory (static) observations (Figure 8, Section 3.3) for low $d_{th}$ (below 0.25). Posteriorly, under-adducted cases feature less vibration, which translates to lower vertical thickness, which is particularly observable in L5 and L7 at a low P_sub_. Overall, under-adducted cases (AL1) feature a lower vertical thickness than the adducted cases (AL2) in no-implant and IM conditions. GM shows a slight decrease in dynamic vocal fold thickness for AL2 and is overall below no implant and IM.

Dynamic vertical thickness reveals that under-adducted folds (AL1) exhibit reduced medial–lateral motion, especially posteriorly. GM results in slightly lower dynamic thickness than IM or no implant under high adduction, suggesting distinct effects on vibratory shaping.

### 3.5. Maximum Divergence Angle

**Figure 10:**
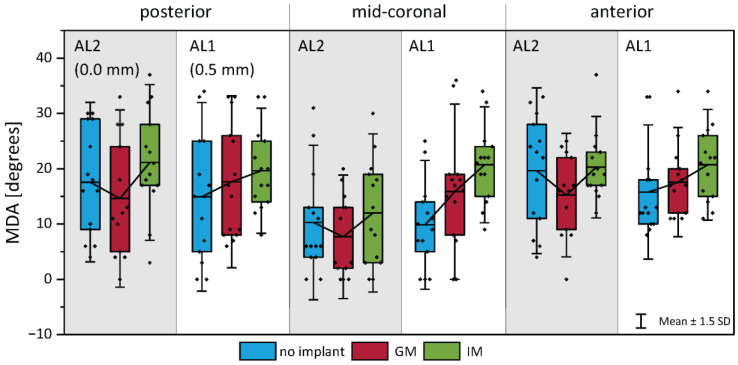
Maximum divergence angle (MDA) of the medial surface during phonation at three anterior–posterior positions: (**left**) posterior, (**center**) mid-coronal, and (**right**) anterior. Bars compare values across adduction levels and implant locations. A higher MDA indicates greater divergent shaping, particularly under infraglottal medialization in under-adducted conditions.

Figure 10 illustrates the maximum divergence angle at the anterior, mid-coronal, and posterior locations under the three implant conditions. Consistent with the hypothesis derived from the VSG measurements, the implant location significantly influenced the MDA during phonation.

In under-adducted conditions (AL1), IM resulted in a more pronounced MDA increase (43% higher than no implant) compared to GM (16%). Conversely, in fully adducted conditions (AL2), where the glottis was already appropriately closed, the addition of an implant did not enhance vibratory parameters and, in several cases, worsened phonation quality (19.6% average reduction in CPP in high P_sub_ cases) as seen in Figure 5. However, even in these conditions, IM generally exhibited a slightly higher MDA compared to GM. Indeed, the average MDA was 24% lower for GM compared to no implant, when IM increased the MDA by 8%.

A statistical analysis supported these observations (Table 3), showing that implant location significantly affected the MDA in under-adducted conditions (AL1). Specifically, IM produced a significantly greater MDA than the no-implant condition at a high subglottal pressure (*p* < 0.05, Cohen’s d = 1.37), with a similar trend at a low pressure (*p* = 0.053, d = 1.27), indicating a strong effect of IM on enhancing vibratory divergence in insufficiently adducted glottal configurations. While IM consistently showed a higher MDA than GM in AL1, this difference was not statistically significant, though effect sizes suggested a moderate to large practical difference. Under fully adducted conditions (AL2), no statistically significant differences were observed between the implant conditions, consistent with the notion that additional medialization provides a minimal benefit when the glottal closure is already adequate.

## 4. Discussion

### 4.1. Acoustic and Flow

The results underscore two critical roles of implants during medialization thyroplasty: (1) reducing the glottal gap by medializing the paralyzed vocal fold, leading to less breathy phonation by decreasing airflow leakage and P_sub_ requirements, and (2) stiffening the paralyzed side, counteracting muscle atrophy, reducing fold bowing, and restoring VSG while maintaining compliance to allow the glottis to open, which is essential for promoting a natural vertical mucosal wave [14].

This effect was most pronounced in under-adducted cases (AL1), where IM led to a better posterior closure and reduced the glottal gap. Improved closure correlates with higher CPP values and a reduced Q, reflecting improved vocal quality and efficiency. In contrast, over-adducted cases (AL2) often resulted in fold over-adduction, degrading phonation quality, and diminishing the benefits of implant placement. This aligns with previous findings where increased medialization improves glottal efficiency and enhances loudness [14,37,38]. The impact of the adduction level was statistically significant on the CPP and Q, whereas the impact of implant location was not, which supports that closing the gap is the most critical role of TT1.

### 4.2. Dynamic Vocal Folds Measurements

While videostroboscopy remains the most widely used tool for clinical glottal assessment, it relies on periodic phonation and provides only 2D surface views. In contrast, 3D measurement techniques have been developed for both hemilarynx and full-larynx models, including digital holography, depth-kymography, laser projection methods, and high-speed stereo imaging systems [2,7,25]. These methods have enabled the more comprehensive characterization of vocal fold dynamics in both experimental and clinical settings.

### 4.3. Static vs. Dynamic Thickness

The current findings show that static vocal fold thickness measured from pre-phonatory geometry does not predict dynamic vibratory behavior. While medialization may increase static thickness (as observed with GM), this does not necessarily translate to enhanced dynamic thickness during phonation. In fact, IM demonstrated greater dynamic thickness despite lower static thickness values, highlighting that vibratory function is governed by how tissue deforms during the phonatory cycle, not by its pre-phonatory shape. It has been hypothesized that increased static thickness may contribute to reducing the phonation threshold pressure by enhancing initial medial contact or stiffness [16,39,40], but it does not determine the distribution or amplitude of vertical mucosal wave motion. These results underscore the importance of dynamic assessment when evaluating medialization outcomes and suggest that optimizing surgical interventions requires consideration beyond static closure alone.

### 4.4. Maximum Divergence Angle

During closing, a divergent shape of the glottis has been shown to improve the efficiency of the voice source [8,9,13]. IM consistently showed a numerically greater MDA than GM, especially in under-adducted conditions (AL1). In adducted cases (AL2), GM even reduced the MDA, showing that a glottal placement may hinder the range of motion of the free margin of the vocal folds. Although this difference between implants was not statistically significant, it supports the hypothesis that implant depth contributes to enhanced divergent glottal shaping, even when glottal closure is already sufficient [37].

### 4.5. Limitations of This Study

While this technique offers superior spatial and temporal resolution and enables the precise measurement of dynamic 3D vocal fold motion, it is currently restricted to ex vivo models and is not applicable in vivo or in clinical settings. Expanding 3D imaging technologies to full-larynx or patient-specific models remains a key direction for future research in voice biomechanics.

Furthermore, the sample size of seven larynges limits the statistical power of our findings. Although the observed trends are consistent with the prior literature, larger datasets are needed to generalize and strengthen the statistical significance of the conclusions [8,9,13,14,33,37,38,41].

### 4.6. Clinical Implications

The findings support the hypothesis that IM may be more effective in cases of UVFP, particularly for enhancing vibratory range and optimizing phonation [14]. By better understanding fold dynamics, surgeons can tailor implant placement to achieve better glottal closure, reduce breathiness, and enhance vocal efficiency. In particular, findings suggest that IM may enhance the dynamic range of motion of the vocal folds.

### 4.7. Future Directions

Further studies should explore the application of 3D-DIC in assessing other vocal fold pathologies and surgical interventions, offering insights into a broader range of clinical scenarios. Characterizing the damping properties of vocal fold tissues through time-resolved DIC could inform the more accurate numerical modeling of vibratory dynamics. Conducting modal analyses of healthy and pathological phonation may also help identify disease-specific vibratory signatures, enhancing diagnosis and treatment planning. Additionally, research should focus on evaluating patient-specific implant designs to optimize surgical outcomes and tailor treatments to individual anatomical and functional needs.

Dynamic changes in glottal thickness under varying phonatory conditions and pressures should be further explored to clarify their role in vocal fold biomechanics and phonatory efficiency. By demonstrating that implant height influences not only static closure but also dynamic vibratory behavior, this study challenges the current one-size-fits-all approach to thyroplasty. These findings may support future clinical trials aimed at personalizing implant placement based on preoperative glottal configuration or predicted vibratory benefits. Incorporating dynamic metrics into surgical planning could ultimately improve voice outcomes in patients with glottal insufficiency.

## 5. Conclusions

This study introduced a method to quantify dynamic vertical thickness and divergence angles from time-resolved 3D medial surface data in an excised hemilarynx model. Results showed that IM increased the glottal divergent shape and dynamic closure more effectively than glottal placement, particularly in under-adducted conditions. These findings underscore the importance of evaluating dynamic behavior, not just static geometry, when optimizing implant strategies for vocal fold medialization.

## Figures and Tables

**Table 1:** Summary of experimental conditions for each larynx.

Subglottal Pressure (P_sub_)	Adduction Level (AL)	Implant Condition
low; high	AL1 (0.5 mm); AL2 (0 mm)	no implant, GM, IM

**Table 2:** Results of a linear mixed-effects model statistical analysis of implant location on CPP and Q.

Group	Contrast	Q		CPP	
		*p*-Value	Effect Size (Cohen’s d)	*p*-Value	Effect Size (Cohen’s d)
AL1-low Psub	GM—IM	0.85	0.29	0.93	−0.2
AL1-low Psub	GM–no implant	0.01	−1.7	1	0.04
AL1-low Psub	IM–no implant	0	−2	0.89	0.24
AL2-low Psub	GM—IM	1	0	0.97	−0.12
AL2-low Psub	GM–no implant	0.83	−0.31	1	0.02
AL2-low Psub	IM–no implant	0.83	−0.31	0.96	0.14
AL1-high Psub	GM-IM	0.98	−0.09	0.96	0.14
AL1-high Psub	GM–no implant	0	−2.09	0.82	0.32
AL1-high Psub	IM–no implant	0	−2	0.94	0.18
AL2-high Psub	GM—IM	0.96	0.15	1	0.02
AL2-high Psub	GM–no implant	0.67	−0.46	0.02	−1.44
AL2-high Psub	IM–no implant	0.5	−0.61	0.02	−1.46

**Table 3:** Results of a linear mixed-effects model statistical analysis of implant location on MDA.

Group	Contrast	MDA	
		*p*-Value	Effect Size (Cohen’s d)
AL1-low Psub	GM—IM	0.55	−0.56
AL1-low Psub	GM–no implant	0.38	0.71
AL1-low Psub	IM–no implant	0.05	1.27
AL2-low Psub	GM—IM	0.77	−0.37
AL2-low Psub	GM–no implant	1	0.03
AL2-low Psub	IM–no implant	0.74	0.4
AL1-high Psub	GM-IM	0.47	−0.63
AL1-high Psub	GM–no implant	0.35	0.75
AL1-high Psub	IM–no implant	0.03	1.37
AL2-high Psub	GM—IM	0.39	−0.73
AL2-high Psub	GM–no implant	0.41	−0.71
AL2-high Psub	IM–no implant	1	0.02

## Data Availability

The data that support the findings of this study are available within the article, as Supplemental Materials, and from the corresponding author upon reasonable request.

## Supplementary Materials

The following supporting information can be downloaded at: https://www.mdpi.com/article/10.3390/bioengineering12060667/s1, Table S1: Phonation trials data.
