# Supplementary material for: Dynamically Quantifying Vocal Fold Thickness: Effects of Medialization Implant Location on Glottal Shape and Phonation
Source: Bioengineering (Basel). 2025 Jun 18;12(6):667. doi: 10.3390/bioengineering12060667 (PMC12189883; doi:10.3390/bioengineering12060667)
Supplement: Supplementary file 1 [file bioengineering-12-00667-s001.zip › bioengineering-3609000-supplementary.pdf]

Supplemental material

Table S1. Phonation trials data.

Larynx	Implant condition	Adduction level	Psub [kPa]	Q [m³/s]	SPL [dB]	Fo [Hz]	frame rate [kHz]	images per cycle
L1	no implant	AL1	*1.376*	*9.17E-04*	73.8	111	1.000	9
L1	no implant	AL1	*1.905*	*1.08E-03*	76.2	114	1.026	9
L1	no implant	AL2	*1.504*	*4.33E-04*	74.3	102	0.952	9
L1	no implant	AL2	*1.865*	*5.64E-04*	74.9	108	1.000	9
L1	glottal	AL1	*1.388*	*7.54E-04*	71.8	106	0.909	9
L1	glottal	AL1	*1.932*	*1.00E-03*	75.0	112	0.952	9
L1	glottal	AL2	*1.438*	*4.32E-04*	72.4	103	0.909	9
L1	glottal	AL2	*1.873*	*5.89E-04*	75.0	112	1.000	9
L1	infra	AL1	*1.358*	*6.69E-04*	73.7	105	0.952	9
L1	infra	AL1	*1.998*	*9.32E-04*	74.5	114	1.053	9
L1	infra	AL2	*1.449*	*3.50E-04*	72.9	93	0.833	9
L1	infra	AL2	*1.867*	*4.78E-04*	75.2	107	1.000	9
L2	no implant	AL1	*1.431*	*9.73E-04*	76.5	107	0.963	9
L2	no implant	AL1	*1.844*	*1.21E-03*	80.5	125	1.165	9
L2	no implant	AL2	*1.469*	*4.25E-04*	72.9	123	1.429	12
L2	no implant	AL2	*2.012*	*6.16E-04*	73.8	157	1.818	12
L2	glottal	AL1	*1.464*	*8.07E-04*	79.1	107	0.925	9
L2	glottal	AL1	*1.955*	*1.05E-03*	82.6	118	1.000	9
L2	glottal	AL2	*1.470*	*4.59E-04*	71.0	106	0.909	9
L2	glottal	AL2	*1.863*	*5.99E-04*	74.8	113	1.053	9
L2	infra	AL1	*1.434*	*7.44E-04*	75.2	105	0.952	9
L2	infra	AL1	*1.902*	*1.02E-03*	80.9	116	1.000	9
L2	infra	AL2	*1.617*	*6.15E-04*	73.1	147	1.333	9
L2	infra	AL2	*1.877*	*6.46E-04*	73.3	119	1.000	9
L3	no implant	AL1	*1.350*	*9.54E-04*	75.5	91	2.000	19
L3	no implant	AL1	*1.926*	*1.40E-03*	79.1	105	2.000	17
L3	no implant	AL2	*1.409*	*5.58E-04*	75.1	80	2.000	22
L3	no implant	AL2	*1.898*	*7.88E-04*	78.1	89	2.000	19
L3	glottal	AL1	*1.414*	*9.58E-04*	76.6	87	2.000	20
L3	glottal	AL1	*1.840*	*1.22E-03*	78.8	94	2.000	18
L3	glottal	AL2	*1.416*	*6.77E-04*	76.0	82	2.000	21
L3	glottal	AL2	*1.870*	*8.77E-04*	78.1	92	2.000	18
L3	infra	AL1	*1.377*	*8.96E-04*	76.2	87	2.000	19
L3	infra	AL1	*1.866*	*1.24E-03*	79.3	95	2.000	18
L3	infra	AL2	*1.400*	*5.56E-04*	75.1	78	2.000	22
L3	infra	AL2	*1.915*	*8.27E-04*	78.0	91	2.000	19
L4	no implant	AL1	*1.446*	*8.02E-04*	71.8	169	2.000	11
L4	no implant	AL1	*1.934*	*1.03E-03*	77.7	180	2.000	10
L4	no implant	AL2	*1.420*	*4.25E-04*	76.3	180	2.000	10
L4	no implant	AL2	*1.848*	*5.51E-04*	80.5	187	2.000	10

L4	glottal	AL1	*1.394*	*6.98E-04*	70.1	169	2.000	11
L4	glottal	AL1	*1.867*	*8.73E-04*	77.6	184	2.000	10
L4	glottal	AL2	*1.403*	*4.11E-04*	76.5	188	2.000	10
L4	glottal	AL2	*1.862*	*5.38E-04*	77.0	195	2.000	9
L4	infra	AL1	*1.386*	*6.86E-04*	72.2	158	2.000	11
L4	infra	AL1	*1.910*	*9.08E-04*	76.0	176	2.000	11
L4	infra	AL2	*1.848*	*4.99E-04*	76.5	196	2.000	9
L4	infra	AL2	*1.451*	*3.88E-04*	75.8	191	2.000	9
L5	no implant	AL1	*1.314*	*7.50E-04*	71.2	-	2.000	-
L5	no implant	AL1	*1.942*	*9.22E-04*	76.2	117	2.000	17
L5	no implant	AL2	*1.378*	*4.60E-04*	71.9	103	2.000	19
L5	no implant	AL2	*1.831*	*5.48E-04*	75.8	110	2.000	17
L5	glottal	AL1	*1.365*	*3.40E-04*	72.6	163	2.000	12
L5	glottal	AL1	*1.768*	*4.79E-04*	74.6	178	2.000	12
L5	glottal	AL2	*1.466*	*2.65E-04*	70.1	188	2.000	11
L5	glottal	AL2	*1.823*	*3.60E-04*	72.2	191	2.000	10
L5	infra	AL1	*1.328*	*4.37E-04*	73.5	123	2.000	16
L5	infra	AL1	*1.772*	*6.29E-04*	77.3	131	2.000	15
L5	infra	AL2	*1.785*	*4.71E-04*	74.5	123	2.000	16
L5	infra	AL2	*1.370*	*3.63E-04*	71.9	120	2.000	16
L6	no implant	AL1	*1.335*	*6.21E-04*	72.1	151	2.000	13
L6	no implant	AL1	*1.882*	*9.26E-04*	76.4	163	2.000	13
L6	no implant	AL2	*1.415*	*3.66E-04*	75.7	155	2.000	13
L6	no implant	AL2	*1.812*	*5.28E-04*	77.9	167	2.000	12
L6	glottal	AL1	*1.306*	*6.07E-04*	73.0	145	2.000	14
L6	glottal	AL1	*1.838*	*8.79E-04*	77.3	157	2.000	13
L6	glottal	AL2	*1.422*	*2.85E-04*	74.0	165	2.000	12
L6	glottal	AL2	*1.790*	*4.07E-04*	76.0	169	2.000	12
L6	infra	AL1	*1.289*	*5.79E-04*	73.1	155	2.000	13
L6	infra	AL1	*1.844*	*8.63E-04*	75.3	165	2.000	12
L6	infra	AL2	*1.773*	*4.57E-04*	76.8	169	2.000	12
L6	infra	AL2	*1.429*	*3.17E-04*	75.1	157	2.000	12
L7	no implant	AL1	*1.412*	*7.63E-04*	74.6	139	2.000	14
L7	no implant	AL1	*1.832*	*9.81E-04*	77.8	152	2.000	13
L7	no implant	AL2	*1.409*	*5.51E-04*	73.6	144	2.000	14
L7	no implant	AL2	*1.838*	*7.32E-04*	77.1	161	2.000	12
L7	glottal	AL1	*1.368*	*6.04E-04*	72.3	139	2.000	14
L7	glottal	AL1	*1.827*	*8.05E-04*	75.5	151	2.000	13
L7	glottal	AL2	*1.422*	*5.06E-04*	72.9	150	2.000	13
L7	glottal	AL2	*1.887*	*6.86E-04*	75.9	163	2.000	12
L7	infra	AL1	*1.379*	*5.82E-04*	73.2	134	2.000	15
L7	infra	AL1	*1.783*	*7.69E-04*	74.8	147	2.000	13
L7	infra	AL2	*1.825*	*5.89E-04*	74.8	161	2.000	13
